# Prevalence of Asymptomatic Malaria Parasitemia among Blood Donors in Cape Coast, Ghana: A Cross-Sectional Study

**DOI:** 10.1155/2023/8685482

**Published:** 2023-01-04

**Authors:** Ato Kwamena Tetteh, Sadick Arthur, Prince Bram, Charles Baffe, Godsway Aglagoh

**Affiliations:** ^1^Metropolitan Hospital, Laboratory Department, P. O. Box 174, Cape Coast, Ghana; ^2^Cape Coast Teaching Hospital, Laboratory Department, P. O. Box CT 1363, Cape Coast, Ghana; ^3^Kasoa Polyclinic, Kasoa, Ghana; ^4^Korle-Bu Teaching Hospital, Laboratory Department, Korle-Bu, Accra, Ghana

## Abstract

**Background:**

Malaria is an important transfusion-associated infection in many parts of the world, particularly in sub-Saharan Africa, where it is endemic. We studied the prevalence of malaria parasites among blood donors in the Cape Coast Metropolitan Area.

**Methods:**

A malaria parasite examination was added to the blood donor screening protocol for 240 voluntary and replacement blood donors (224 males and 16 females) between December 2020 and July 2021.

**Results:**

Overall, 2.5% (6/240) had *Plasmodium falciparum* trophozoites detected in their blood sample. The remaining had no parasites detected. Four of the 148 who passed the blood donor screening tests were infected. The remaining two with malaria parasites failed one screening test. These included one donor with “hepatitis *B* + *P. falciparum*” and another with “syphilis + *P. falciparum*” parasite coinfection. All blood donors who had malaria parasites detected in their blood were males. Most donors, 45.8% (110/240), were in the 26–35 age group, with the highest prevalence of 1.3% (3/240). Blood group O was predominant (75.0%, 180/240), followed by B (12.9%, 31/240), A (11.3%, 27/240), and AB (0.8%, 2/240). All malaria parasites detected were among individuals with blood group O. Moreover, 96.3% (231/240) were rhesus-positive and had the highest prevalence of 2.1% (5/240).

**Conclusions:**

Screening of blood donors in Ghana does not include malaria, although there is the potential for transmission through blood products. Malaria transmission via blood transfusion remains an issue of public health concern, as indicated in the results of this current study. We recommend studies on malaria prevention, pretransfusion and posttransfusion, and pathogen reduction technology.

## 1. Introduction

The World Malaria Report estimates 241 million malaria cases in 2020 and 227 million in 2019 [1]. Malaria is known to cause 1.5–2.7 million deaths worldwide, particularly in Africa [2]. It is estimated that over 95% of malaria cases and 96% of malaria deaths are concentrated in Africa [3]. It is a disease with very high morbidity and mortality rates in Africa [4]. Malaria claimed the lives of an estimated 445,000 people worldwide, with Africa accounting for roughly 91% of the total [3]. It is one of the vector-borne diseases that can be transmitted through blood transfusion, aside from chagas, toxoplasmosis, leishmaniasis, babesiosis, and microfilariasis [5].

The causative organism for malaria is the *Plasmodium* species, which are *P. falciparum, P. vivax, P. ovale*, *P. malariae,* and *P. knowlesi*. All four human malaria parasites (*P. falciparum, P. vivax, P. ovale,* and *P. malariae*) could be transmitted through blood transfusion [6]. *Plasmodium falciparum* is responsible for the severest form of malaria and is the most prevalent in sub-Saharan Africa [4]. Some *Plasmodium* species can survive in stored blood for seven to 40 days, depending on the species.

Blood donors are not routinely screened for malaria parasites before donation, even though the World Health Organization recommends that all donor blood be tested for malaria in most malaria-endemic countries in sub-Saharan Africa [7]. Thus, there is an increased potential for malaria parasite transmission to blood recipients and clinical diseases [8].

Blood transfusions can be lifesaving for individuals who have lost large volumes of blood. A blood transfusion may be required in serious accidents, gynaecological haemorrhages, surgery, stem cell transplants, symptomatic anaemia, and cancer [9]. Although blood is used to save lives, it could do more harm than good if not screened thoroughly to prevent the transmission of diseases [10]. The most affected groups include children under five years, pregnant women, victims of serious blood loss in road traffic accidents, and immunosuppressive patients [7]. Blood and its products are essential in emergencies for every healthcare system. However, using blood products may be complicated by the risks of adverse immunological reactions and transmission of other blood-borne pathogens [11].

Since blood donors may have malaria parasites, there could be a risk of transmission to vulnerable blood recipients. The intraerythrocytic stage of the parasite can be transmitted by transfusion of any blood component containing infected cells. An initial report on malaria resulting from a blood transfusion was published in 1911 [12]. Some clinical manifestations of malaria include headache, generalized body pain, especially in the back and limbs, anorexia, nausea, chills, and fever [11].

The National Blood Transfusion Service of Ghana stated in 2010 that the country requires 250,000 pints of blood annually. While there are some studies regarding malaria parasitemia in donor blood around the middle belt of Ghana [12–14], it is uncertain how many transfused blood units contain malaria parasites in the southern part of Ghana. In sub-Saharan Africa, malaria parasitemia in blood donors ranges from 0.6 to 50% [4]. We conducted this preliminary study to add to existing data and determine the occurrence of malaria parasitemia among blood donors in Cape Coast, the capital of the Central Region of Ghana.

## 2. Materials and Methods

### 2.1. Study Site

The Cape Coast Metropolitan Hospital (CCMH) is a 98-bed government hospital located at 24 Beulah Road (5.1021°N, 1.2597°W), Cape Coast, Central Region. The CCMH is equipped with a district-level medical laboratory, obstetrics and gynaecology, paediatrics, male/female wards, antenatal and postnatal care, public health, nutrition, herbal medicine units, a theatre, and a mortuary. The recent COVID-19 pandemic has led to the addition of a new COVID-19 ward, a COVID-19 PCR testing centre, and an oxygen gas plant. The hospital's OPD attendance is about 100–150 patients during the day's peak hours (8 : 00 am–2 : 00 pm). Between July and August, when it typically rains, is when malaria cases are at their highest in Cape Coast.

**Figure 1:**
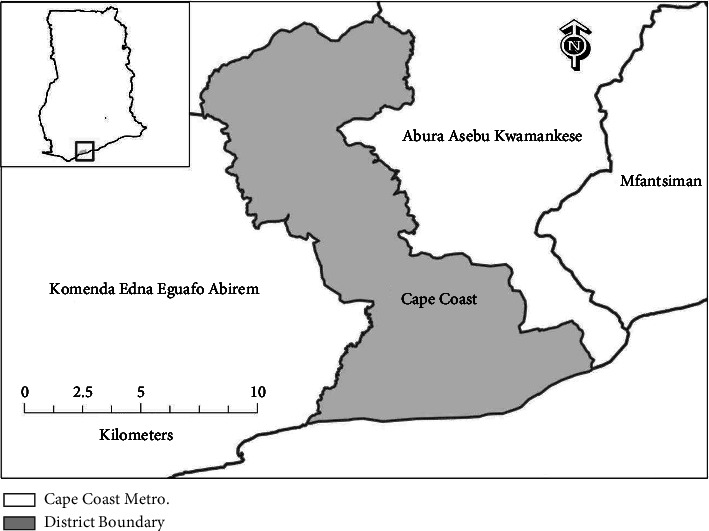
Map of the Cape Coast (credit: Ing. Dr. Charles Gyamfi, Department of Civil Engineering, Kwame Nkrumah University of Science and Technology, Kumasi, Ghana).

Cape Coast is the regional capital of the Central Region and shares borders with the Gulf of Guinea to the south, Komenda-Edina-Eguafo-Abbrem (KEEA) Municipal to the west, Abura-Asebu-Kwamankese (AAK) District to the east, and Twifo-Hemang Lower Denkyira District to the north (Figure 1). Facts from the 2021 population and housing census show that the Metropolis has a population of 189,925 people, with 92,790 men and 97,135 women.

### 2.2. Inclusion Criteria

To be included, participants (volunteers, predeposit, and replacement donors) had blood pressure measurements between 60–90 and 90–140 mmHg (diastolic/systolic) and body weight greater than or equal to 50 kg. Adequate haemoglobin concentration level (>12.5 g/dl for females and >13.5 g/dl for males) was also considered an important inclusion criterion. The participants were visibly healthy asymptomatic individuals who showed no signs of malaria, such as fever in the cold or sweating, headache, and clinical signs of anaemia, joint pain, generalized weakness, and vomiting. Both first-time and repeat donors were included. However, repeat donors should not have donated blood in less than the past three months.

### 2.3. Exclusion Criteria

All blood donors who did not satisfy the abovementioned descriptions and had tattoos and other visible skin rashes or bruises and responded to having drunk alcohol in the past 12–24 hours or engaged in direct smoking of any form were automatically excluded. Also excluded from the study were those donors who took any antimalarial drugs within the last two weeks before the blood test. Participants who were taking drugs for high blood pressure were excluded. Though systolic/diastolic measurements may be normal, the bleeding process may trigger a hypertension crisis. Females who were menstruating or breastfeeding were not included. Those who did not consent at any stage of the screening process were also excluded.

### 2.4. Sampling Method

This study design was cross-sectional. The participant selection was passive and purposeful, and samples were collected daily from December 2020 through July 2021, until the sample size was achieved. These blood donors comprised 224 males and 16 females between the ages of 16 and 45.

#### 2.4.1. Sample Size Estimation

Our sample size was based on a similar study and population setting conducted by Ofosu *et al.* at the Asamankese Government Hospital, Eastern Region, Ghana, where a sample size of 240 was estimated [4]. Sample size, *n*=*N*/[1+*N*(*e*^2^)], where *N* is the approximate number of donors screened within the year and *e* is the margin of error (5%). In our laboratory, an average of 580 blood donors is screened annually. Therefore, *n*=580/[1+580(0.05^2^)]=236.73 〜240 blood donors.

### 2.5. Sample Collection and Testing

The venepuncture technique was used. Venous blood was collected using 5 ml disposable syringes fitted with needles. The blood was dispensed into 5 ml EDTA tubes and mixed gently.

Laboratory analysis determined the donors' blood group and serological tests (hepatitis B, hepatitis C, syphilis, and HIV). Thick and thin films were prepared with 6 *µl* and 2 *µl* of blood on grease-free labelled slides using a smooth-edged slide spreader. The thin film side of each slide was fixed in absolute methanol and air-dried. All smears were then stained with 10% Giemsa for 10 minutes. The slides were washed with buffered water and air-dried on drying racks. The sexual and asexual stages of the *Plasmodium* parasites were identified using 100× light microscope magnification. The tile method was used to determine a blood donor's ABO group. Three drops each of anti-sera A (anti-A), anti-B, and anti-D were applied to a clean and dry white ceramic tile using a Pasteur pipette. Equal amounts of thoroughly mixed donor blood were mixed with the antisera over a 1.5 cm diameter area on the tile. After 2 minutes of incubation at room temperature, the mixtures on the tile were examined for agglutination. Data on the age and sex of each donor were documented during blood collection.

### 2.6. Statistical Analysis

Data entry and analysis were carried out using Statistical Package for the Social Sciences (IBM® SPSS, Version 25.0, https://www.ibm.com), software for Windows. The data extracted were analysed and presented using descriptive statistics according to age, sex, blood group, and malaria parasitemia outcome. Binary logistic regression analysis on the malaria microscopy test outcome (dependent variable) and the independent variables listed above did not yield significant associations (*p* > 0.05). The numbers in the subgroups were not enough to test for associations. Therefore, the table for the regression analysis was not included in the results.

### 2.7. Ethical Consideration

Blood donors whose data were used in this article voluntarily agreed to have their anonymized information recorded through informed consent. The hospital granted permission for this study, provided that the data were only used for research and learning purposes, would help improve the management of patients in the future, would cause no harm under the Declaration of Helsinki (1964), and was part of the requirements for allied health professionals working in the hospital's laboratory to renew licenses. Participants screened out of the blood donation process were provided adequate support through the hospital's counselling unit and treated.

## 3. Results

### 3.1. Demographics

Out of the 240 blood donors, 224 (93.3%) were males and 16 (6.7%) were females (Table 1). The mean age of the blood donors was 27.95 ± 6.14 (mean ± SD) with a range of 17–45 years. Most of the donors, 110 (45.8%), were in the age group 26–35, followed by 16–25 (42.1%), and then 36–45 (12.1%). All six (2.5% positive) individuals were males. Among the age groups, 26–35 years recorded the highest prevalence of malaria parasitemia 3 (1.3%), followed by 36–45 years (0.8%), and then 16–25 years (0.4%). The commonly occurring ABO blood group was blood group O 180 (75%), while blood group AB was the least 2 (0.8%). All positive malaria cases were among the blood group O category. Most of the populations were rhesus-positive (96.3%, 231/240). Five malaria-positive cases were identified from rhesus-positive donors (Table 1).

Six of the 240 individuals examined were infected, indicating an overall prevalence of 2.5%. These six (2.5%) individuals were infected with *Plasmodium falciparum*. Of the 240 individuals, 148 (61.7%) qualified (passed all screening tests) for donation, while 92 (38.3%) were disqualified (low haemoglobin concentration or failed one screening test) based on the four serological tests performed. Four (1.7%) of the qualified blood donors and two (0.8%) of the disqualified had malaria parasites (Table 2).

Most disqualified individuals were reactive for syphilis (22.1%) (53/240), followed by hepatitis B (13.3%) (32/240). One blood donor (0.4%) tested positive for HIV. One of the positive donors for hepatitis B was also coinfected with malarial parasites. One blood donor who was reactive to syphilis had malaria parasites (Table 3).

## 4. Discussion

Malaria, in general, is not only a serious threat because posttransfusion malaria can exacerbate recipients' already poor health, but it can also be fatal. Therefore, the need for effective donor selection guidelines cannot be overstated [15]. This study sought to determine the prevalence of malaria infection among blood donors in Cape Coast. Blood donors were individuals who had visited the laboratory to either donate blood for a relative, replace blood for a relative, or in walk randomly to donate blood voluntarily. The overall prevalence of malaria parasitemia among blood donors was 2.5%, while that among those who passed the screening test and donated was 1.7%.

All malaria-positive blood donors in this study were infected with *Plasmodium falciparum,* which is not a surprising or solitary finding. This finding is congruent with other studies where *P. falciparum* was either the predominant or the only *Plasmodium* species detected [11]. *Plasmodium falciparum* is the most endemic malaria species in Ghana and sub-Saharan Africa, and it is recognized as the severest form of malaria and is responsible for most malaria deaths [12, 16, 17].

The prevalence in this study is comparable to another conducted among blood donors at the Asamankese Government Hospital in Ghana, which was 2.7% [4]. As well, our prevalence is lower when compared to similar studies such as 4.1% in Maiduguri, Nigeria [18], 6.8% in Zaria, Nigeria [19], 7.5% in Kaduna, Nigeria [11], and 13.0% in Kumasi, Ghana [12]. This study's prevalence was lower when compared to some other studies, such as 28.0% in Jos [20] and Lagos, Nigeria [21], 30.2% in Nnewi, Nigeria [22], 51.5% in Abakaliki, Nigeria [2], and 67.5% in Port Harcourt, Nigeria [16]. Our findings are also in contrast to what has been reported in some countries, such as 0.76% in Dhaka City, Bangladesh [23], 0.58% in Peshawar, Pakistan [24], and 0.00% on microscopic examination in India [10]. The different geographical locations and levels of endemicity could account for the variations in the proportions.

Typically, more males than females volunteer to donate blood. Most females do not meet the haemoglobin concentration requirement for donating blood. This phenomenon, therefore, accounts for the large difference between male and female participants in this study. The high male participation in blood donation could account for the higher prevalence of malaria in males (2.5%) than in females (0.0%). The higher prevalence rate observed for the males in this study is in harmony with Muntaka and Opoku-Okrah [12] and Mbanugo and Emenalo [25]. Still, it differs from Otajevwo's [26] findings and Vlassoff and Bonilla's [27], where females had a higher *Plasmodium* parasite infection rate than males. The highest prevalence was among those 26–35 years old (1.3%, 3/240), as observed by Okocha et al. [22].

Although the polymerase chain reaction (PCR) and enzyme-linked immunosorbent assay (ELISA) methods for all these diseases are available commercially, they are only available in tertiary care health institutions in Ghana. As laboratories in Ghana continue to participate in the SLMTA (Strengthening Laboratory Management Through Accreditation) programme [28], introduced in 2009, challenges with infrastructure and upgrading medical laboratories with modern equipment to facilitate the use of more sensitive testing will be available in resource-limited settings as well.

The most common blood group was “O,” and the least common was “AB.” Our study is consistent with most reports showing the dominance of blood group O [20, 29] among blood donors. All positive malaria cases were among blood group O individuals. To a large extent, this outcome is biased because blood group O is the most sought-after; therefore, we perceive that individuals with blood group O mostly volunteer to support their friends and relatives. This reason could account for the observation in this study. Our opinion differs from that of Abah and Joe-Cliff [7]. They hypothesized that blood group O might protect against severe malaria and even prevent people with blood type O from dying. They concluded from their study that this reasoning could illustrate why blood group O is the predominant group infected with malaria parasites. Rhesus-positive donors accounted for 96.3% of the total participants and constituted 2.1% (5/240) of the total prevalence. The rhesus-negative participants had a prevalence of 0.4% (1/240).

In 2005, Kitchen et al. published a review on *Plasmodium* species in 100 transfusion-transmitted malaria (TTM) case reports with varying occurrences of the following: *P. falciparum* (45%), *P. malariae (30%), P. vivax* (16.0%), *P. ovale* (4%), *P. knowlesi* (2%), and a mixed infection comprising *P. falciparum* and *P. malariae* (1%) [30]. Natural infection through the bite of an infected female *Anopheles* mosquito and TTM vary markedly, with the former going through the liver stage, which stimulates host defense cells against malaria parasites and gives the naive host time to produce a much more directed protective immunity. The risk of complications rises when infected blood transfusions directly release malaria parasites into the recipient's bloodstream, inhibiting the activation of innate immunity [31]. Thus, malaria presents a serious complication in blood transfusions despite being rare. The presence of asymptomatic blood donors who are primarily “partially immune” and have very low parasite loads is the main issue with TTM. Due to this, donor screening using thick and thin blood smears, the gold standard for diagnosing malaria, might not be the most suitable [32].

Although not sufficiently supported and implemented, exciting novel technologies are on the horizon in western nations. The most prevalent and virulent form of malaria in sub-Saharan Africa, *P. falciparum*, is highly sensitive to inactivation by photochemical treatment with amotosalen and long-wavelength ultraviolet light [33, 34]. These pathogen reduction measures have the potential to lower TTM.

### 4.1. Strength of the Study

1. Tests were performed immediately with the screening sample and not from stored blood to ensure that the parasite and red blood cell biological features did not deteriorate.

### 4.2. Limitations of the Study

1. Although three WHO-trained malaria microscopists thoroughly examined blood films in our laboratory, much more sensitive methods are required for comparison. These methods will enable us to screen blood that the “gold standard” might miss.
2. Sampling was purposeful; hence, we perceive that most blood donors knew their blood group and might have had experience with blood donation. We envisage a different outcome in a nonhealth facility or mobile session blood donation exercise.
3. To realize our turnaround time for the blood donation, we could not administer questionnaires to investigate other relevant information. We relied on routine hospital records for information.

## 5. Conclusion

A 2.5% malaria parasite prevalence among asymptomatic blood donors suggests a risk to blood recipients, particularly those susceptible to malaria. This outcome suggests the possibility of transfusion-related malaria. In the regular assessment of potential blood donors, we propose that all blood earmarked for transfusion be screened for malaria parasites and labelled negative or positive. Malaria-infected blood may be useful to patients whose circumstances permit malaria prevention therapy. The reason is that malaria is curable, and rejecting low-intensity malaria-infected blood might exacerbate the country's present donor blood shortage. There is, however, an urgent need, in the short term, to determine prophylactic and treatment protocols for posttransfusion malaria in endemic regions, including Ghana.

## Figures and Tables

**Table 1:** Distribution of malaria parasite infection among blood donors.

Variables	No. of examined (%)	No. of infected (%)^*∗*^
*Gender*		
Male	224 (93.3)	6 (2.5)
Female	16 (6.7)	0 (0.0)

Age group		
16–25	101 (42.1)	1 (0.4)
26–35	110 (45.8)	3 (1.3)
36–45	29 (12.1)	2 (0.8)

*ABO group*		
A	27 (11.3)	0 (0.0)
B	31 (12.9)	0 (0.0)
AB	2 (0.8)	0 (0.0)
O	180 (75.0)	6 (2.5)

*Rhesus group*		
Rhesus positive	231 (96.3)	5 (2.1)
Rhesus negative	9 (3.8)	1 (0.4)

^*∗*^ *Plasmodium falciparum* intensity ranged from 40 to 800 trophozoites/µL of blood.

**Table 2:** Prevalence of malaria parasitemia in examined donors.

Category^*∗*^	No. of examined (%)	No. of infected (%)
Qualified to donate	148 (61.7)	4 (1.7)
Disqualified to donate	92 (38.3)	2 (0.8)
Total	240 (100)	6 (2.5)

^*∗*^Qualified to donate: passed all screening tests (negative hepatitis B, hepatitis C, syphilis, and HIV serological tests). Disqualified: low haemoglobin concentration or positive for one screening test.

**Table 3:** Prevalence of malaria parasitemia in examined disqualified donor blood.

Categories	No. of positive/reactive (%)	No. of infected with malaria parasites (%)
Hepatitis B	32 (13.3)	1 (0.4)
Hepatitis C	6 (2.5)	0 (0.0)
Syphilis	53 (22.1)	1 (0.04)
HIV	1 (0.4)	0 (0.0)
Total	92 (38.3)	2 (0.8)

## Data Availability

All the data in this study are included within the article.
